# Flocculation Characteristics and Dewatering Performance of Bored Pile Waste Slurry with Different Flocculants

**DOI:** 10.3390/ma19153299

**Published:** 2026-08-04

**Authors:** Shouju Miao, Jun Chen, Zhihai Zhang, Shuirong Gui, Ling Zhou, Shengjie Liu

**Affiliations:** 1 Jiangxi Provincial Communications Investment Group Co., Ltd., Nanchang 330000, China; 2 Jiangxi Provincial Highway and Bridge Engineering Co., Ltd., Nanchang 330008, China; 3 School of Civil Engineering and Architecture, East China Jiaotong University, Nanchang 330013, China; 4 College of Civil and Transportation Engineering, Hohai University, Nanjing 210098, China

**Keywords:** bored pile waste slurry, flocculation law, dewatering performance, zeta potential, specific filtration resistance, solid–liquid separation

## Abstract

**Highlights:**

**What are the main findings?**

- Anionic polyacrylamide (APAM) showed superior low-dosage adaptability for bored pile waste slurry treatment;
- APAM at 0.2–0.3% achieved efficient sedimentation and dewatering performance;
- Organic polymer flocculants improved particle aggregation more effectively than polymeric aluminum chloride (PAC).

**What are the implications of the main findings?**

- The results support efficient in situ treatment of bored pile waste slurry;
- Optimized APAM dosage can reduce chemical consumption and treatment costs;
- The findings provide guidance for efficient and controlled disposal of engineering waste slurry.

**Abstract:**

Bored pile waste slurry generated during bridge substructure construction exhibits high water content and stable colloidal properties, resulting in difficult solid–liquid separation and severe environmental risks. To improve solid–liquid separation and facilitate efficient disposal, this study systematically investigated the flocculation and dewatering performance of the slurry using three typical flocculants (Anionic polyacrylamide (APAM), cationic polyacrylamide (CPAM), and polymeric aluminum chloride(PAC)) through sedimentation tests, supernatant purification monitoring, particle size analysis, zeta potential measurements, and specific filtration resistance tests. The results show that flocculant type and dosage significantly affect treatment efficiency. Organic flocculants outperform inorganic PAC in both particle aggregation and dewatering improvement. Among them, APAM exhibits a prominent low-dose advantage; at an optimal dosage of 0.2–0.3% (corresponding to a pure reagent dosage of 10–15 mg/L based on wet slurry), it effectively reduces the slurry water content and lowers supernatant suspended solids below the 50 mg/L discharge standard within 80 min. Mechanistically, PAC provides primarily electrostatic neutralization, whereas APAM and CPAM achieve composite flocculation through charge neutralization, adsorption bridging, and sweep capture. Benefiting from efficient low-dose neutralization and long-chain bridging effects, APAM significantly reduces filtration resistance and forms stable flocs. Overall, an APAM dosage of 10–15 mg/L is the optimal scheme for in situ treatment of bored pile waste slurry, providing technical support for the efficient management and controlled disposal of engineering slurry under similar geological conditions.

## 1. Introduction

The rapid expansion of global transportation infrastructure has driven continuous scale growth in bridge engineering construction. Bored pile foundations are widely adopted in bridge engineering, owing to their high bearing capacity and strong geological adaptability; they have become the dominant construction method for bridge substructures [1,2,3]. However, bored pile drilling operations generate substantial volumes of waste slurry: typically 3–5 times the volume of the drilled hole. The waste slurry is characterized by ultra-high water content, strong fluidity, and fine particle gradation. Improper disposal poses serious potential threats to ecological environments and urban operational safety [4,5,6]. To avoid high treatment costs, some construction units have engaged in illegal discharge of waste slurry into urban rivers and municipal pipe networks [7,8]. Such illegal acts not only degrade water quality and raise riverbed elevations but also cause pipeline blockages, significantly increasing the operational load of municipal drainage and sewage treatment systems. In addition, direct landfill of high-fluidity waste slurry may induce secondary geological hazards such as ground subsidence.

Current mainstream waste slurry treatment technologies face prominent technical bottlenecks and limitations. Ex situ centralized treatment is constrained by high transportation costs, low treatment efficiency, leakage risks, and secondary pollution problems [9,10,11]; moreover, slurry transport vehicles frequently interfere with urban traffic operations. Natural sedimentation can achieve partial volume reduction, but the fine colloidal particles in waste slurry exhibit strong suspension stability, resulting in an extremely slow sedimentation rate [12,13,14]. This approach requires large-scale sedimentation ponds and long treatment cycles, which severely restricts construction progress and substantially increases project costs. Therefore, it is urgent to develop economical and efficient in situ volume reduction and dewatering technologies for bored pile waste slurry.

Chemical flocculation–dewatering technology has been widely verified as an efficient method for the rapid volume reduction in waste slurry, as flocculants can disrupt colloidal stability, promote the aggregation of fine suspended particles into large flocs, and accelerate solid–liquid separation [15,16,17,18]. Recent studies have further shown that slurry treatment performance depends strongly on flocculant type and formulation. Xu et al. compared a newly synthesized Fe-PAM with different ionic PAMs and found that the combination of charge neutralization and long-chain adsorption bridging markedly accelerated flocculation and improved filtration performance [19]. Lu et al. reported that combined PAC and APAM treatment produced better filtration and water-discharge performance than APAM alone in dredged slurry [20]. More recently, Zhang et al. demonstrated that a ternary (P(AM-AMPS-DMDAAC) (PAAD))PAAD/PAC/CaCl_2_ system outperformed standalone PAAD, commercial CPAM, and binary combinations in dewatering field-collected pile-foundation sludge [21]. These findings indicate that slurry treatment efficiency is governed not only by dosage but also by the ionic character, molecular structure, and combination mode of flocculants. Nevertheless, the physical and chemical properties of pile-foundation waste slurry vary significantly across regions due to differing geological conditions, leading to notable differences in slurry composition and characteristics. Existing studies have largely focused on specific slurry types or newly developed/composite flocculation systems, while systematic comparisons of conventional flocculants that link flocculation–sedimentation dynamics with dewatering performance remain limited [22,23,24]. In practical engineering, the selection of flocculant type and dosage is often based on empirical judgment, which frequently leads to suboptimal treatment outcomes and poor applicability in real projects.

To address existing research gaps, this study systematically investigates the laws of flocculation and sedimentation alongside the solid and liquid separation efficiency of engineering pile foundation waste slurry treated with three typical flocculants (CPAM, APAM, and PAC). Dynamic monitoring with multiple parameters was conducted throughout the process to elucidate the influence mechanism of flocculant type and dosage on the physicochemical properties of the slurry. The intrinsic evolution laws of flocculation and sedimentation were revealed, and specific resistance to filtration tests were performed to quantitatively evaluate the solid and liquid separation performance. Consequently, a relationship between flocculant dosage and dewatering performance was established. Through a comparative analysis of the flocculation and dewatering effects, the optimal flocculant type and operational parameters suitable for regional pile foundation waste slurry were determined, along with the corresponding enhancement mechanisms. The findings provide theoretical support and technical guidance for efficient dewatering, volume reduction, and controlled disposal of bored pile waste slurry, thereby facilitating the standardized application of slurry treatment technologies. Significantly, by addressing challenges associated with region specific slurries, this research provides essential quantitative data for optimizing traditional flocculant application, establishing a scientific basis for standardized treatment in similar geological contexts.

## 2. Materials and Methods

### 2.1. Materials

The waste bored pile slurry used in this test was collected from a highway expansion and reconstruction project in Jiangxi Province, China. The basic physical properties of the original slurry were tested in accordance with national standard test methods: water content of 165.9% (oven drying method), density of 1.28 g/cm^3^ (pycnometer method), and pH value of 8.35 (pH meter method). The specific mineral composition of the slurry is shown in Table 1.

Three typical flocculants were selected for the test, including two organic flocculants and one inorganic flocculant: anionic polyacrylamide (APAM), cationic polyacrylamide (CPAM), and polymeric aluminum chloride (PAC). The APAM used has a molecular weight of 1.8 × 10^7^ and an anion degree of 32%; the CPAM used has a molecular weight of approximately 2.0 × 10^7^ and a cation degree of 40%; and the PAC used has a basicity of approximately 70%, an Al_2_O_3_ content of 30%, and a polymeric aluminum salt form. PAC was prepared as a solution with a concentration of 1 g/L, while CPAM and APAM were prepared at a concentration of 5 g/L.

### 2.2. Experimental Methods

#### 2.2.1. Settling Column Test

The bored pile waste slurry was used directly in its original state as received, without undergoing drying or the addition of water. The mixed suspension was then slowly poured into a 500 mL graduated cylinder. The cylinder was gently inverted five times to ensure uniform dispersion of the flocculant within the slurry system, after which it was placed statically for natural sedimentation. The elevation of the clear liquid–slurry interface was dynamically monitored in real time, and the sedimentation curve of the slurry was plotted. After sedimentation, the volume of the consolidated sediment was measured, and the water content of the sediment was calculated accordingly. All test groups were repeated three times under the same dosage condition, to ensure the reliability and repeatability of the test results.

#### 2.2.2. Suspended Solids Test

The flocculant dosage was defined as the mass ratio of flocculant solution to raw slurry. Specifically, three dosage levels of 0.1%, 0.3%, and 0.5% were applied for CPAM and APAM, and 0.6%, 1.2%, and 1.8% for PAC. Each flocculant solution was mixed with raw slurry via a two-stage stirring procedure, including rapid mixing followed by slow stirring to accomplish flocculation. Thereafter, the treated slurry was transferred to gradu-ated cylinders for static sedimentation over 80 min, and supernatant samples were col-lected at 0, 20, 40, 60, and 80 min during the settling process. For suspended solid (SS) measurement, microporous filter membranes were placed in weighing bottles, oven-dried at 103–105 °C for 30 min, cooled in a desiccator, and repeatedly dried and weighed to achieve a constant mass. The pre-weighed membranes were installed on a vacuum filtra-tion device and fully wetted prior to testing. After filtration, the membrane retaining sus-pended solids was transferred back to the original weighing bottle, dried at 103–105 °C for 1 h, cooled to room temperature, and weighed repeatedly. The change in SS concentration was calculated using the standard Equation (1):

(1) $$\mathrm{SS}=\frac{(A-B)\times 10^{6}}{V}$$

where *SS*—suspended solids concentration in water, mg/L; *A*—weight of suspended solids + filter membrane + weighing bottle, g; *B*—weight of filter membrane + weighing bottle, g; *V*—sample volume, mL.

#### 2.2.3. Slurry Particle Size Test

The particle size distribution of the flocculated slurry was measured using a Malvern Mastersizer 3000 laser particle size analyzer (Malvern Panalytical, Malvern, UK). The raw slurry sample was first shaken to disperse any pre-existing aggregates and ensure homogeneity. Subsequently, predetermined dosages of the flocculants were added to induce flocculation. The flocculated slurry was allowed to stand for 8 h to facilitate sedimentation, after which the supernatant was removed. The settled sediment was then dried in an oven to ensure complete moisture removal. Finally, the particle size distribution of the dried floc samples was analyzed using the wet laser diffraction method, and the average value of multiple tests was taken as the final result.

#### 2.2.4. Zeta Potential Test

The zeta potential of the slurry samples was measured using a Malvern Zetasizer Ultra nanoparticle potential analyzer(Malvern Panalytical, Malvern, UK). After the flocculation reaction, the slurry was allowed to stand for a short period to allow large flocs to settle. An aliquot of the upper suspension, which still contained fine particles and micro-flocs, was carefully extracted for measurement. It is crucial to note that the sample was not filtered, as the zeta potential measurement requires the presence of suspended particles. The test was conducted at room temperature, and the average value of multiple tests was taken as the final result.

#### 2.2.5. Specific Resistance to Filtration Test

The sludge specific resistance to filtration (SRF) was adopted to characterize the filtration and dewatering performance of the flocculated slurry. SRF is a core index that reflects the filtration resistance of unit mass of sludge under unit filtration area and unit pressure, and it can directly characterize the difficulty of slurry pressure-filtration dewatering. A lower SRF value indicates better filtration performance and higher dewatering efficiency of the slurry. SRF measurements were performed on the flocculated slurry samples using a conventional Buchner funnel vacuum-filtration setup, which consisted of a filter holder and a graduated receiver cylinder. A constant vacuum pressure of 80 kPa was maintained throughout the test. The volume of filtrate was collected in the graduated cylinder and recorded continuously every 10 s. The SRF value was calculated using the standard Equation (2):

(2) $$SRF=\frac{2PA^{2}b}{\mu \omega },$$

where *P* is the applied vacuum pressure (kg/m^2^); *A* is the effective filtration area (m^2^); *b* is the slope of the linear fitting curve of filtration time t and filtrate volume *V* (×10^12^ s/m^6^); *μ* is the dynamic viscosity of filtrate (kg·s/m^2^); and *ω* is the dry solid mass per unit volume of slurry, i.e., filter cake weight corresponding to unit filtrate volume (kg/m^3^).

## 3. Results

### 3.1. Slurry Settling Law

**Figure 1:**
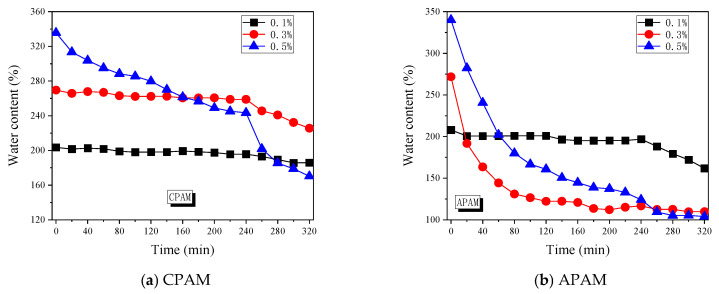

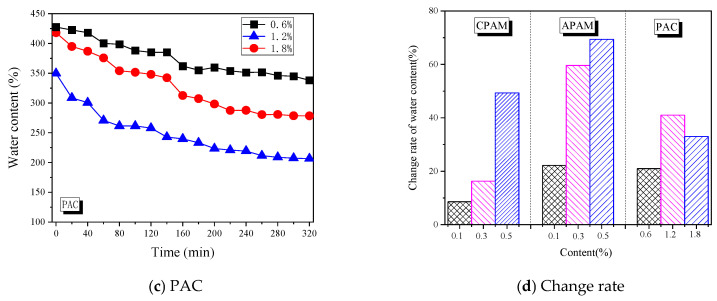
Time-dependent changes in the water content of bored pile waste slurry treated with (**a**) CPAM (0.1%, 0.3%, and 0.5%), (**b**) APAM (0.1%, 0.3%, and 0.5%), and (**c**) PAC (0.6%, 1.2%, and 1.8%), together with (**d**) a comparison of the corresponding water-content change rates.

The dynamic variation in slurry water content under different dosages of APAM, CPAM, and PAC flocculants is presented in Figure 1, which clearly demonstrates a significant interactive effect between flocculant type and dosage on the sedimentation kinetics of the slurry. It should be noted that the raw slurry possessed an ultra-high initial water content of 165.9% and a bulk density of 1.28 g/cm^3^, with abundant fine colloidal particles uniformly dispersed in the system. This structural characteristic endowed the slurry with strong suspension stability, leading to extremely slow natural sedimentation and difficult solid–liquid separation without flocculant intervention.

For CPAM (Figure 1a), at the lowest dosage of 0.1%, the water content exhibited minimal variation, remaining relatively stable at approximately 200%. This limited flocculation activity directly reflects the inherent strong suspension stability of the slurry, indicating that a low dosage of CPAM is insufficient to break the stable colloidal matrix. However, increasing the dosage to 0.3% and 0.5% resulted in a noticeable reduction in water content, with the 0.5% dosage showing the most pronounced decline in the later stages of sedimentation. This suggests that CPAM requires a moderate dosage to overcome the slurry’s initial physicochemical stability and enhance sedimentation efficiency.

APAM exhibited distinct performance characteristics (Figure 1b). At 0.1% dosage, although the initial water content was higher, the water content decreased rapidly within the first 40 min, reflecting superior sedimentation kinetics at low concentrations. This rapid response indicates that APAM’s molecular structure is highly effective at destabilizing the uniformly dispersed colloidal particles, rapidly transforming the stable suspension into settleable flocs. When the dosage was increased to 0.3% and 0.5%, the initial water content was slightly lower, but the overall decline rate was marginally slower; nonetheless, the final water content at both dosages approached 120%, demonstrating effective dewatering. This highlights APAM’s ability to achieve rapid and efficient solid–liquid separation even at low dosages, making it a technically and economically favorable option for treating this specific type of engineering waste slurry.

In contrast, PAC showed a strong dependence on dosage (Figure 1c). At dosages of 0.6%, 1.2%, and 1.8%, the final water content progressively decreased with increasing dosage. Notably, at the 1.8% dosage, which achieved the fastest dehydration rate, the reduction in water content from the initial to the final state reached 100%, indicating maximum water release. This indicates that PAC requires a relatively high dosage to achieve optimal sedimentation and dewatering efficiency, as lower dosages are likely ineffective. The dosage sensitivity of PAC is evident, with performance improving significantly only at higher concentrations.

The change rate of water content (Figure 1d) further corroborates these observations. APAM at 0.1% dosage exhibited the highest initial change rate, highlighting its rapid capability to break the slurry’s suspension stability at low concentrations. PAC, on the other hand, showed a higher change rate at higher dosages, consistent with its dosage-dependent performance. CPAM displayed the lowest change rate across all tested dosages, indicating a more gradual process in overcoming the colloidal barriers.

Overall, the results suggest that APAM offers superior performance at low dosages, with rapid initial water content reduction and effective dewatering. CPAM maintains stability at low dosages but is outperformed by APAM and PAC at higher dosages. PAC, while requiring higher dosages, achieves the best results at 1.8% in terms of final water content. Because the raw slurry is incapable of meeting the requirements for direct discharge due to its physicochemical defects, these findings emphasize the importance of selecting the appropriate flocculant type and dosage to optimize sedimentation efficiency. APAM emerges as a preferred candidate for on-site treatment of bored pile waste drilling mud due to its balanced performance and economic viability, providing a scientific basis for optimizing flocculant application in engineering sludge disposal.

### 3.2. Suspended Solids of Settled Slurry

A large number of fine colloidal suspended particles exist in untreated waste slurry, which are the primary cause of high turbidity and excessive suspended solids (SS) in the supernatant. These fine particles feature small particle size, large specific surface area, and stable dispersion characteristics. Electrostatic repulsion and hydration film effects maintain their long-term suspension in aqueous solution, resulting in poor natural sedimentation performance and excessive supernatant SS concentration that far exceeds the national discharge standard of 50 mg/L. The weakly alkaline environment of raw slurry further stabilizes the colloidal suspension system of fine particles, exacerbating the difficulty of supernatant purification.

**Figure 2:**
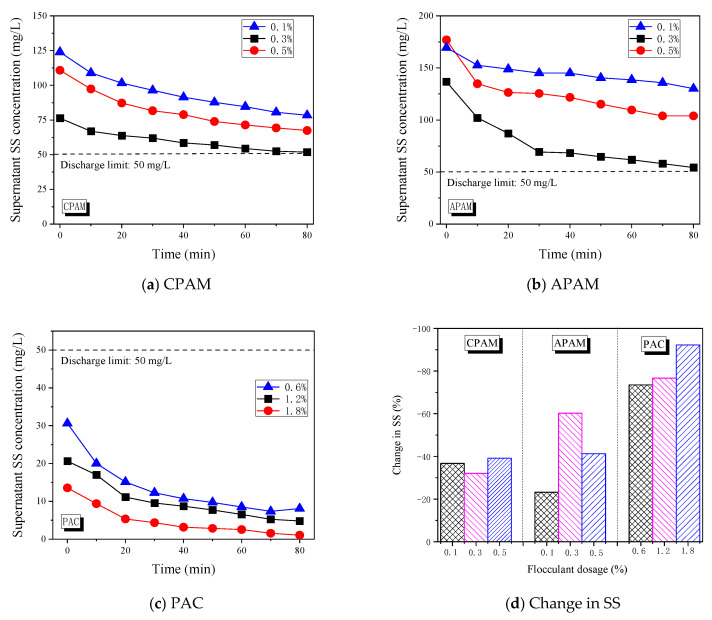
Time-dependent changes in supernatant suspended solids (SS) for bored pile waste slurry treated with (**a**) CPAM (0.1%, 0.3%, and 0.5%), (**b**) APAM (0.1%, 0.3%, and 0.5%), and (**c**) PAC (0.6%, 1.2%, and 1.8%), together with (**d**) a comparison of the corresponding SS change rates over the 80 min settling period.

The SS content of the flocculated slurry was measured and compared against the tertiary effluent standard of 50 mg/L stipulated in the Urban Wastewater Treatment Plant Pollutant Discharge Standard (GB 18918-2002) [25]. The results are shown in Figure 2.

As illustrated in Figure 2a–c, the temporal variations in SS content were monitored for CPAM, APAM, and PAC under their respective dosage ranges: 0.1–0.5% for CPAM and APAM, and 0.6–1.8% for PAC. The results demonstrate that both flocculant type and dosage significantly influence SS removal efficiency and settling kinetics.

For CPAM (Figure 2a), the lowest dosage of 0.1% yielded the highest initial SS content, with a gradual decline over 80 min to approximately 50 mg/L. Increasing the dosage to 0.3% and 0.5% accelerated SS reduction, with the 0.5% dosage achieving the most pronounced decline, approaching the 50 mg/L limit by 80 min. This indicates that CPAM requires moderate dosages to enhance SS removal, while low dosages exhibit limited effectiveness.

APAM exhibited superior performance at lower dosages (Figure 2b). At 0.1%, despite an initial SS content of 180 mg/L, the SS decreased rapidly within the first 40 min, falling below 50 mg/L by 80 min. Even at higher dosages, APAM maintained efficient SS removal, with final concentrations consistently below the standard. This highlights APAM’s ability to achieve rapid and effective solid–liquid separation at low-to-moderate dosages.

In contrast, PAC showed a strong dosage dependence (Figure 2c). With dosages of 0.6%, 1.2%, and 1.8%, SS content progressively decreased with increasing dosage, with the 1.8% dosage achieving near-complete removal by 80 min. PAC’s performance improved markedly only at higher concentrations, underscoring its sensitivity to dosage and the need for elevated dosages to meet the effluent standard. Among all tested conditions, 1.8% PAC yielded the lowest residual SS concentration and therefore exhibited the best SS removal performance when this index was considered independently.

The change rate of SS content (Figure 2d) further corroborates these trends. APAM at 0.1% dosage exhibited the highest initial change rate, reflecting its rapid flocculation capability at low concentrations. PAC, conversely, showed higher change rates at elevated dosages, consistent with its dosage-dependent performance. CPAM displayed the lowest change rate across all tested dosages, indicating a more gradual SS removal process.

Combined with the water-content evolution results in Figure 1, the SS variation curves in Figure 2 indicate a synergistic relationship between flocculation efficiency and mud–water separation. APAM, particularly at 0.2–0.3%, promoted rapid initial settling and stable floc formation, resulting in effective suspended-solid removal and improved dewatering performance. PAC achieved the lowest residual SS at 1.8%, confirming that it was the best-performing flocculant in terms of SS removal alone. However, its relatively high dosage demand limits its economic advantage. CPAM demonstrated moderate sensitivity to dosage changes and produced a more gradual SS removal process.

These findings emphasize that judicious selection of flocculant type and dosage is critical for optimizing both SS removal efficiency and mud dewatering performance. Accordingly, PAC should be regarded as the best option for maximizing SS removal under the tested conditions, whereas APAM remains the preferred overall candidate based on the combined performance across sedimentation, particle aggregation, and dewatering indicators. The observed interactive effects also provide a scientific basis for optimizing flocculant application in engineering sludge management.

### 3.3. Slurry Particle Size Distribution

**Figure 3:**
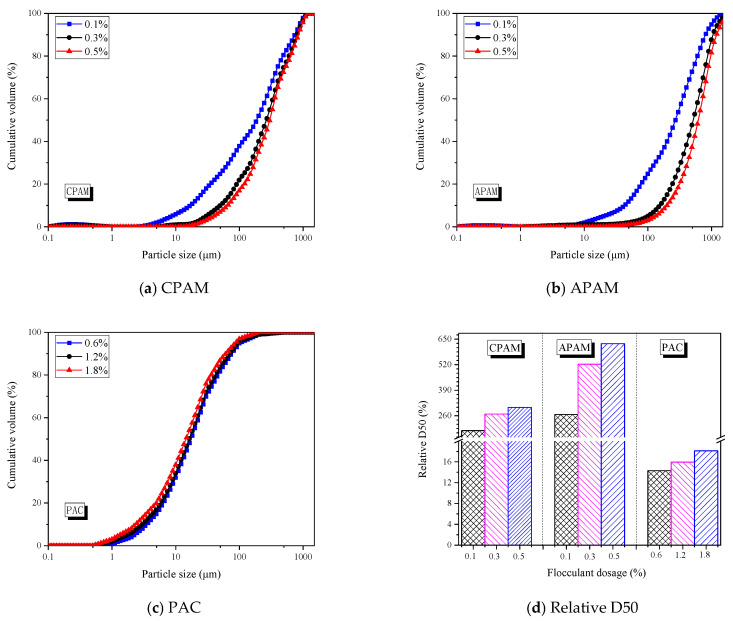
Volume-based cumulative particle-size distributions of bored pile waste slurry treated with (**a**) CPAM (0.1%, 0.3%, and 0.5%), (**b**) APAM (0.1%, 0.3%, and 0.5%), and (**c**) PAC (0.6%, 1.2%, and 1.8%), and (**d**) comparison of D50 changes under the tested dosage conditions.

To analyze the slurry particle-size distribution and flocculation performance of CPAM, APAM, and PAC, the results from Figure 3a–d are discussed, integrating curve trends, characteristic size variations, and flocculation mechanisms to elucidate differences between organic and inorganic flocculants.

For CPAM-treated mud, the cumulative curves exhibited a dose-dependent rightward shift relative to the unmodified sample, indicating enhanced aggregation and coarsening of particles. At a dosage of 0.1%, the median particle size (D50) increased from approximately 10 μm to approximately 15 μm, and the 90% passing size (D90) rose from approximately 60 μm to approximately 80 μm. As the dosage increased to 0.3% and 0.5%, the curves further shifted toward larger particle sizes, with D50 reaching approximately 20 μm and D90 approximately 100 μm at 0.5%. However, the growth rate of particle size slowed markedly beyond 0.3%, as evidenced by the minimal difference between the 0.3% and 0.5% curves.

APAM-treated mud demonstrated superior coarsening performance compared to CPAM at identical dosages. The cumulative curves for 0.1%, 0.3%, and 0.5% APAM were more right-shifted than those of CPAM, with D50 reaching approximately 25 μm and D90 approximately 120 μm at 0.3% values significantly larger than those of CPAM at the same dosage. Like CPAM, APAM exhibited an optimal dosage threshold, beyond which additional flocculant provided marginal gains due to the saturation of active sites on particle surfaces.

In contrast to organic flocculants, PAC-treated mud showed negligible changes in particle-size distribution. The cumulative curves overlapped with the unmodified sample, and key characteristic sizes remained stable: the 10% passing size (D10) stayed around 1–2 μm, D50 was stably maintained at 10–15 μm, and D90 ranged only between 60 and 80 μm. Even at the highest dosage, PAC could not alter the original fine-grained gradation of the mud, underscoring its limited efficacy in regulating particle-size distribution.

The D50 change rate further validated performance differences among the three flocculants. APAM at 0.1% dosage showed the highest initial change rate, reflecting rapid flocculation at low concentrations via multi-mechanism particle aggregation. PAC exhibited higher rates at elevated dosages due to enhanced electrostatic screening, but required higher doses for minimal effects. CPAM had the lowest rate across all dosages, indicating gradual solid suspension removal from its saturation-prone mechanism. The results clearly demonstrated distinct flocculation mechanisms for organic (CPAM, APAM) and inorganic (PAC) agents. These findings provided a scientific basis for flocculant selection in solid–liquid separation, with APAM emerging as the preferred agent for efficient, low-dose treatment of bored pile waste drilling mud, offering a practical strategy for compliant and high-efficiency disposal.

### 3.4. Slurry Zeta Potential

The raw waste slurry presents a stable negative zeta potential ranging from −18 mV to −22 mV. The negative surface charge of slurry particles generates persistent electrostatic repulsion between adjacent fine particles, inhibiting particle collision and aggregation and forming a thermodynamically stable colloidal suspension system. This electrical property constitutes the core chemical mechanism for the long-term stability and poor sedimentation performance of waste slurry. Different types of flocculants can effectively alter the surface charge characteristics of slurry particles and break colloidal stability, thereby achieving solid–liquid separation.

**Figure 4:**
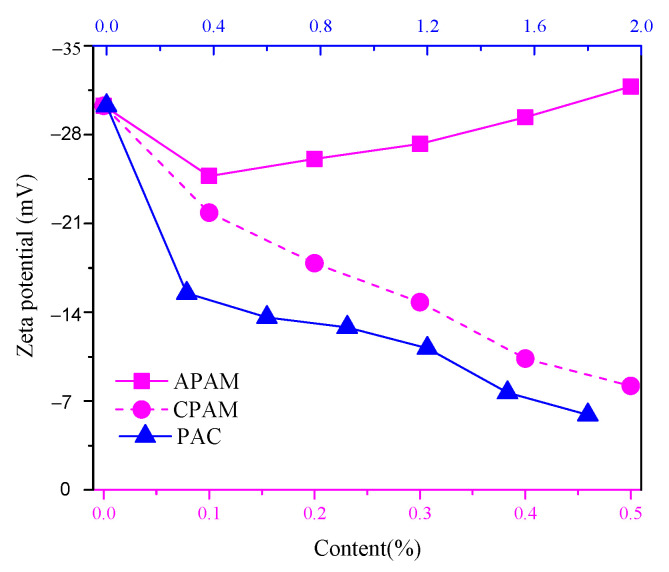
Zeta potential of bored pile waste slurry as a function of APAM, CPAM, and PAC dosage under the tested conditions. Measurements were conducted at room temperature.

As shown in Figure 4, the zeta potential of the waste slurry exhibited distinct trends with increasing flocculant dosage. APAM displayed a downward trend, with the zeta potential becoming progressively more negative as the dosage increased. This behavior aligns with its anionic nature, indicating an increased negative surface charge following APAM adsorption. In contrast, CPAM shifted the zeta potential toward less negative values with increasing dosage, reflecting charge neutralization by its cationic groups. PAC exhibited a rapid and pronounced increase in zeta potential, stabilizing between –4 and –7 mV at a dosage of 0.2–0.5%, which reflects near-complete charge neutralization by the cationic inorganic species. However, the absence of a long-chain structure limits the ability of PAC to form large flocs via adsorption-bridging, thereby explaining its inferior performance compared to APAM.

The increasingly negative zeta potential of APAM, combined with its long-chain molecular structure, indicates that its superior flocculation performance is primarily driven by adsorption-bridging and sweep capture rather than charge neutralization. While CPAM shifts the zeta potential toward less negative values through charge neutralization, its overall flocculation and dewatering performance remains inferior to that of APAM under the tested conditions. The rapid shift in PAC toward a near-neutral potential indicates strong charge neutralization; however, without the bridging capability of organic polymers, it produces only marginal particle-size coarsening. Overall, APAM emerges as the optimal flocculant for treating bored pile waste slurry, offering high efficiency at low dosages, rapid sedimentation, and excellent dewatering. In contrast, PAC and CPAM exhibit reduced efficacy due to limited bridging capabilities and weaker overall performance, respectively.

The fundamental reasons underlying these distinct trends lie in the molecular architectures and interfacial interaction mechanisms of the three flocculants. Although APAM introduces negative charges upon adsorption—thereby lowering the zeta potential—its long-chain structure can span the electric double layer (EDL) to form a robust three-dimensional network between particles via hydrogen bonding. This predominantly generates large flocs through adsorption-bridging and sweep capture mechanisms. Conversely, CPAM relies primarily on its cationic groups to compress the EDL for electrostatic neutralization. Nevertheless, constrained by its molecular weight, its spatial bridging capacity is restricted, resulting in weaker floc stability. PAC rapidly neutralizes charges and destabilizes colloids via high-valence cations; however, lacking an organic long-chain structure, it fails to cross-link particles into large aggregates, forming only minute, compact clusters. Therefore, the interplay between charge characteristics and molecular configuration fundamentally dictates the divergent flocculation behaviors observed among these three flocculants.

### 3.5. Slurry Specific Resistance

The untreated raw waste slurry has an extremely high specific resistance to filtration (SRF) The ultra-fine particle gradation and stable colloidal structure result in dense hydration films adsorbed on particle surfaces and tiny pore channels in filter cakes during filtration. These characteristics induce tremendous filtration resistance, low filtration rate, and inferior solid–liquid separation and dewatering performance, which severely restrict the efficient pressure filtration and dewatering of engineering waste slurry.

**Figure 5:**
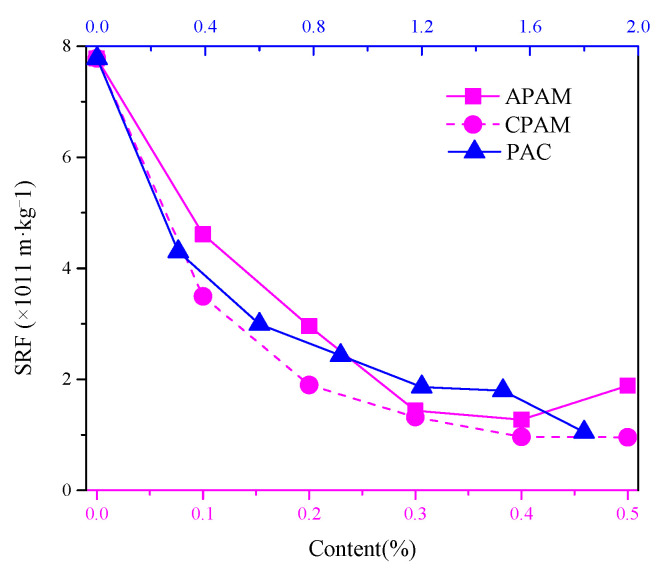
Specific resistance to filtration (SRF) of bored pile waste slurry treated with APAM, CPAM, and PAC at different dosages under a constant vacuum pressure of 80 kPa.

Prior to the SRF test, flocculated slurry samples were prepared by mixing the waste bored pile mud with predetermined dosages of the selected flocculants: APAM (0.1%, 0.3%, and 0.5%), CPAM (0.1%, 0.3%, and 0.5%), and PAC (0.6%, 1.2%, and 1.8%). Specifically, a designated volume of the waste mud was placed in a 1000 mL beaker, followed by the addition of the corresponding flocculant solution. The mixture was then rapidly stirred at 120 r/min for 1 min to ensure uniform dispersion and facilitate the formation of stable flocs within the slurry system. As shown in Figure 5, the effects of the three flocculants on the specific resistance to filtration (SRF) of bored pile waste drilling mud exhibited significant differences. After comprehensive performance comparison, APAM emerged as the optimal choice.

APAM achieved a rapid SRF reduction at low dosages, with the value dropping from an initial ~8.0 × 10^−11^ m/kg to below 2.0 × 10^−11^ m/kg within the first 0.1% addition, followed by stabilization. This performance stems from its strong adsorption-bridging capacity: as an organic anionic flocculant, APAM’s long-chain molecular structure promotes multi-point adsorption and interparticle bridging, forming large, compact flocs that reduce the sludge’s specific surface area and thus accelerate solid–liquid separation. Its high efficiency at low dosages also lowers treatment costs, meeting economic requirements.

Although PAC achieved a rapid SRF reduction, it required higher dosages to reach comparable levels with APAM. As an inorganic flocculant, PAC realizes charge neutralization via high-charge-density Al^3+^, but its lack of long-chain molecular structure limits bridging capacity, necessitating larger dosages to form stable flocs and increasing treatment costs.

CPAM exhibited the slowest SRF reduction and showed a subtle rebound at dosages above 0.3%, indicating insufficient bridging strength and partial dispersion of aggregated flocs, which led to an SRF increase. This behavior is consistent with the zeta potential results, reflecting CPAM’s limited adsorption-bridging efficiency under the tested conditions and its poorest solid–liquid separation performance.

Owing to its low-dosage rapid SRF reduction, stable floc formation, and excellent solid–liquid separation performance, APAM emerged as the optimal flocculant for treating bored pile waste drilling mud. Its combination of high efficiency and economic efficiency effectively meets engineering requirements.

## 4. Conclusions

This study systematically investigated the flocculation behaviors, solid–liquid separation characteristics, and dewatering performance of bored pile waste slurry treated with APAM, CPAM, and PAC. The synergistic relationships among flocculant type, dosage, colloidal stability, particle aggregation, and dewatering efficiency were clarified. The main concise conclusions are drawn as follows:

(1) Flocculant type and dosage significantly govern the treatment efficiency. APAM demonstrates superior low-dosage adaptability, achieving rapid sedimentation and effective dewatering at 0.2–0.3%; CPAM requires a moderate dosage (≥0.5%) to produce noticeable effects; and PAC demands a substantially higher dosage (≥1.2%) to achieve comparable performance. At the optimal dosage, APAM reduces the slurry water content to approximately 120% and lowers the supernatant suspended solids below the 50 mg/L discharge standard within 80 min.
(2) The three flocculants operate through distinctly different mechanisms. PAC provides only single electrostatic neutralization; although it rapidly shifts the zeta potential toward neutral values, its lack of a long-chain structure limits particle bridging and floc growth. CPAM achieves charge neutralization and moderate bridging, yet its floc stability remains restricted. APAM, benefiting from its anionic long-chain molecular structure, enables a composite mechanism of charge neutralization, adsorption-bridging, and sweep capture, forming large and stable flocs that significantly improve particle size distribution and solid–liquid separation.
(3) The SRF test quantitatively confirms the dewatering advantage of APAM. At only 0.1% dosage, APAM rapidly reduces the SRF from an initial ~8.0 × 10^−11^ m/kg to below 2.0 × 10^−11^ m/kg, whereas PAC requires much higher dosages to reach a comparable level, and CPAM exhibits the slowest reduction with a rebound at dosages above 0.3%. This indicates that APAM can substantially lower filtration resistance and treatment costs at low chemical consumption.
(4) The established dose–effect relationship and the identified flocculation mechanisms provide a scientific basis for construction units to select flocculant types and dosages according to regional slurry characteristics. It should be noted that the recommended optimal scheme of APAM at 0.2–0.3% for in situ treatment of bored pile waste slurry in this work is solely based on laboratory-scale technical flocculation and dewatering performance.

## Figures and Tables

**Table 1:** The specific mineral composition of the slurry.

Component	SiO_2_	Fe_2_O_3_	Al_2_O_3_	MgO	Na_2_O	CaO	ZnO	K_2_O
Content/%	62.8	5.6	17.3	1.8	4.3	2.8	0.01	3.2

## Data Availability

The original contributions presented in the study are included in the article, further inquiries can be directed to the corresponding author.

## Abbreviations

The following abbreviations are used in this manuscript:

APAM	Anionic polyacrylamide
CPAM	Cationic polyacrylamide
PAC	Polymeric aluminum chloride
SRF	Specific resistance to filtration
SS	Suspended solids
D50	Median particle size
D90	Particle size below which 90% of particles are distributed
